# Can Topical Insect Repellents Reduce Malaria? A Cluster-Randomised Controlled Trial of the Insect Repellent *N,N*-diethyl-*m*-toluamide (DEET) in Lao PDR

**DOI:** 10.1371/journal.pone.0070664

**Published:** 2013-08-14

**Authors:** Vanessa Chen-Hussey, Ilona Carneiro, Hongkham Keomanila, Rob Gray, Sihamano Bannavong, Saysana Phanalasy, Steven W. Lindsay

**Affiliations:** 1 Faculty of Infectious and Tropical Diseases, London School of Hygiene and Tropical Medicine, London, United Kingdom; 2 Centre for Malariology, Parasitology and Entomology, Ministry of Health, Vientiane, Lao People's Democratic Republic; 3 Population Services International Laos, Vientiane, Lao People's Democratic Republic; 4 School of Biological and Biomedical Studies, Durham University, Durham, United Kingdom; Kenya Medical Research Institute (KEMRI), Kenya

## Abstract

**Background:**

Mosquito vectors of malaria in Southeast Asia readily feed outdoors making malaria control through indoor insecticides such as long-lasting insecticidal nets (LLINs) and indoor residual spraying more difficult. Topical insect repellents may be able to protect users from outdoor biting, thereby providing additional protection above the current best practice of LLINs.

**Methods and Findings:**

A double blind, household randomised, placebo-controlled trial of insect repellent to reduce malaria was carried out in southern Lao PDR to determine whether the use of repellent and long-lasting insecticidal nets (LLINs) could reduce malaria more than LLINs alone. A total of 1,597 households, including 7,979 participants, were recruited in June 2009 and April 2010. Equal group allocation, stratified by village, was used to randomise 795 households to a 15% DEET lotion and the remainder were given a placebo lotion. Participants, field staff and data analysts were blinded to the group assignment until data analysis had been completed. All households received new LLINs. Participants were asked to apply their lotion to exposed skin every evening and sleep under the LLINs each night. *Plasmodium falciparum* and *P. vivax* cases were actively identified by monthly rapid diagnostic tests. Intention to treat analysis found no effect from the use of repellent on malaria incidence (hazard ratio: 1.00, 95% CI: 0.99–1.01, p = 0.868). A higher socio-economic score was found to significantly decrease malaria risk (hazard ratio: 0.72, 95% CI: 0.58–0.90, p = 0.004). Women were also found to have a reduced risk of infection (hazard ratio: 0.59, 95% CI: 0.37–0.92, p = 0.020). According to protocol analysis which excluded participants using the lotions less than 90% of the time found similar results with no effect from the use of repellent.

**Conclusions:**

This randomised controlled trial suggests that topical repellents are not a suitable intervention in addition to LLINs against malaria amongst agricultural populations in southern Lao PDR. These results are also likely to be applicable to much of the Greater Mekong Sub-region.

**Trial Registration:**

This trial is registered with number NCT00938379

## Introduction

At present global malaria prevention efforts are focused on the distribution of long-lasting insecticidal nets (LLINs) and indoor residual spraying [1]. LLINs are the current best practice and protect from mosquito biting indoors at night, but where vectors bite outdoors or early in the evening their effectiveness may be reduced. The major Afro-tropical malaria vector *Anopheles gambiae* typically feeds at night and indoors, making LLINs a perfect intervention tool against this species. However the major vectors in Southeast Asia and South America commonly feed outdoors [2]–[4]. Also of concern is the threat of increased outdoor biting as a result of either species shifts or behaviour change in response to insecticide use [5]–[7]. Therefore there is a growing need for intervention tools that can protect from outdoor biting.

Topical repellents have the potential to reduce vector contact in this setting. Field trials in Thailand and Malaysia show di-ethyl-*N,N-*toluamide (DEET) concentrations of 15–20% decrease mosquito biting by over 83% [8]–[10]. However few trials have been able to demonstrate an effect on malaria transmission by the use of insect repellent. On the Thai-Myanmar border, pregnant women were given either thanaka (a traditional cosmetic derived from *Limonia acidissima*) mixed with DEET or thanaka alone. Although a 29% reduction in *P. falciparum* was observed, the transmission level was too low for this to be statistically significant [11]. Similarly, in Afghanistan low malaria rates meant a 45% reduction in malaria (96% of cases were *P. vivax*) observed in people using a repellent soap containing 20% DEET and 5% permethrin was non-significant [12]. In Ecuador and Peru a village randomised trial of repellent soap found no reduction in malaria compared to untreated controls [13]. Nonetheless, a 56% reduction in the odds of *P. falciparum* infection was found in Pakistan amongst households using repellent soap compared to those using a placebo, although no effect was found for *P. vivax* infections [14]. Households using 30% *p*-Menthane-3,8-diol (PMD, a repellent derived from lemon eucalyptus, *Eucalyptus maculata citriodon*) in Bolivia had an 80% lower incidence of *P. vivax* [15]. There was also an 82% reduction in *P. falciparum* but case numbers were too low to reach significance. A number of common problems have affected the results of these trials. Lower than expected malaria rates have resulted in insufficient sample sizes and non-significant reductions. Compliance is also very important, since repellent requires application every few hours it is easy to forget, lose and even apply in insufficient doses. The inconsistency of these results means that it is not yet established whether the use of insect repellent can reduce malaria infection.

The highest malaria incidence rates in Lao PDR are found in Attapeu and Sekong provinces along the southern borders with Cambodia and Vietnam [16]. *Plasmodium falciparum* causes almost 97% of cases and *P. vivax* the remainder [17]. Village based surveys in Attapeu have found increased malaria risk to be associated with sleeping without a bed net and visits to the forest [18], [19]. Unusually for Southeast Asia where young men are most at risk of malaria infection, studies have not previously found a gender bias in Lao PDR, although young children are the group with the highest rates of malaria [18]–[20]. The main vector is *Anopheles dirus* which is strongly associated with forests and is frequently found biting outdoors [21], [22]. Biting time varies depending on sibling species, whilst for most peak biting occurs from 21.00–02.00 h, other species start feeding at 18.00 h [21], [23]. *Anopheles minimus* and *An. maculatus* are also important vectors and are similarly found biting outdoors in the early evening [4], [19], [24]–[26].

The aim of this trial was to determine whether using a topical 15% DEET repellent, established by landing catches to reduce mosquito biting by 98.9%, would reduce malaria incidence against exophagic vectors amongst rural populations in southern Lao PDR using LLINs.

## Methods

The protocol for this trial and supporting CONSORT checklist are available as supporting information; see Checklist S1 and Protocol S1.

### Study area

**Figure 1:**
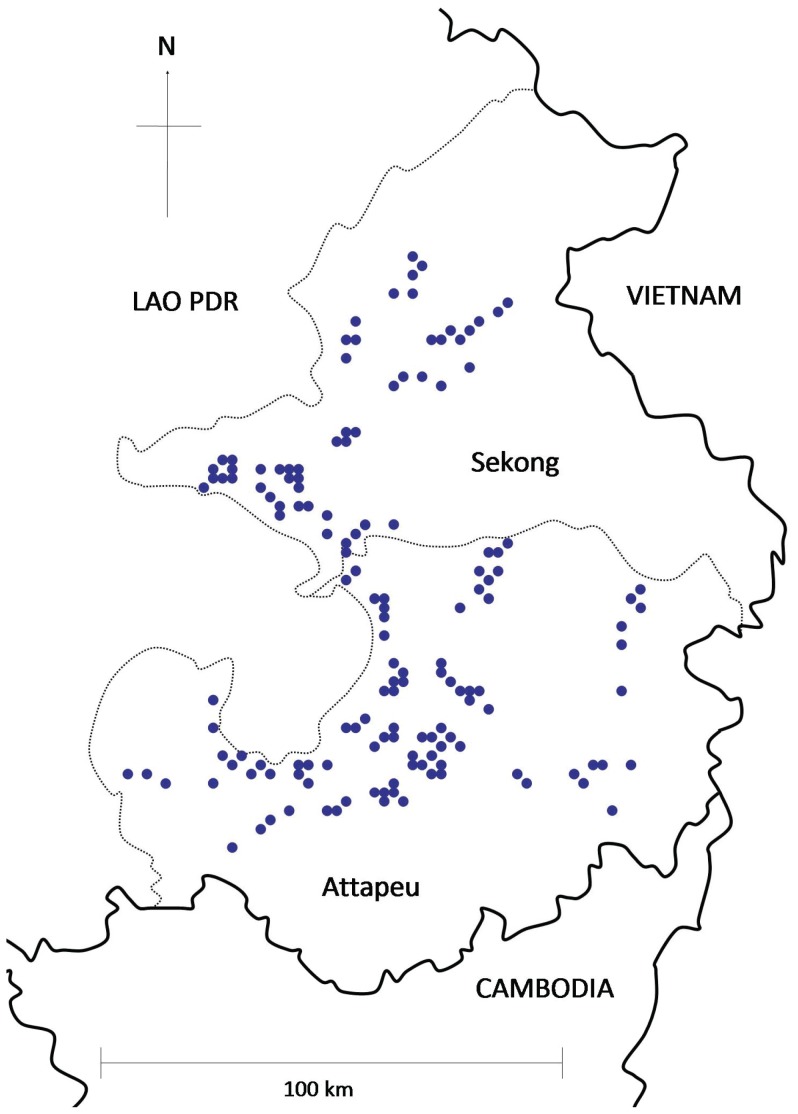
Location of study villages in Attapeu and Sekong provinces in Southern Lao PDR.

Attapeu and Sekong are the most south-eastern provinces in the Lao PDR, sharing borders with Vietnam and Cambodia (Figure 1). The Annamite mountains run along the eastern Vietnam border and 60% of Attapeu Province is mountainous. The mountains are covered with dense rainforest, contrasting with the open canopy dry forest on the plains. The wet season is usually from April to October, followed by a cool dry season from November to January and a hot dry season from February to March. Rice farming is the main economic activity with 57% of Attapeu's population being farmers and 71% in Sekong [27]. The malaria situation in Lao PDR is similar to that across the mainland Southeast Asia; low overall, but a severe problem in forested border areas. Within Lao PDR, it is the southern provinces that are most affected by malaria, with *P. falciparum* parasite rate in Attapeu and Sekong about twice as high as the national average [27]. *Plasmodium falciparum* is found in about 80% of cases, and *P. vivax* in most of the rest [19], [20], [22], [26]. The most important malaria vectors are *An. dirus*, *An. minimus* and *An. maculatus* [25], [28], and all feed early and outside implying that they will be less affected by conventional control methods such as LLINs [4], [19], [23], [25], [29]–[31]. However although these behaviours may reduce the effectiveness of LLINs in reducing malaria transmission, non-use of a bed net is still associated with malaria in Lao PDR [18], [19]. Current policy in the country is for the entire population at risk (estimated to be 70% of the country) to receive LLINs [17]. In addition free diagnosis and treatment with artemisinin combined therapy (ACT) has been implemented to poor populations. Resistance to artemisinin has not yet been detected here [32].

### Recruitment

Households were recruited from 126 villages; 72 in Attapeu Province and 54 in Sekong Province. Participants were primarily rural agricultural workers that often work and sleep overnight away from the village during the wet season. Eligible households needed five volunteers aged 6–60 years. Study households also had to be separated by at least 10 m to prevent diversion of biting from repellent users to placebo users. District health staff obtained written informed consent from all participants or the carers of participants aged under 18 years. Baseline information was collected on age, gender and household wealth indicators.

A household-cluster randomised design was chosen so that members of the same household were assigned to the same treatment. This avoided accidental mix-up of treatments, but also prevented potential diversion of biting from repellent users to placebo users in close proximity. This effect has been demonstrated over 1 m and could lead to the overestimation of the protection given by repellents [33]. The only reliable epidemiological data from the study area prior to the start of the trial was 38% prevalence of *P. falciparum* from village surveys [34]. Previous trials of topical repellent have shown an 80% reduction in clinical malaria due to *P. vivax* and a 44% reduction in *P. falciparum* infection [14], [15]. A 50% reduction in clinical malaria was therefore considered to represent a useful malaria intervention in this setting. An initial sample size of 500 households per arm was sufficient to detect a 50% reduction in clinical malaria associated with repellent use at 90% power and 95% significance [35]. This was based on an estimated pre-trial incidence of 2–6%. However, a malaria incidence of only 0.7% was recorded in the first year of data collection. Malaria cases also showed clustering at the village level, but only one household had more than 1 case of *P. falciparum* supporting the assumption that cases were not over-dispersed by household. Therefore a coefficient of variation of 0.25 was used. The spatial heterogeneity at village level howerer, underlined the importance of stratifying randomisation by village. Sample sizes were recalculated and a sample size of 633 households per arm was found necessary to detect a 50% reduction in malaria incidence with 95% level of significance at 80% power. This was adjusted to approximately 800 households per arm to account for non-compliance and loss to follow up.

### Intervention

Households were randomised to one of the two treatment arms using equal groups allocation which was stratified by village. Heads of households picked treatment codes out of a bowl. A 15% DEET lotion was provided to households in one treatment arm and the remainder received a placebo lotion (both supplied by SCJohnson, Racine, USA). Adult participants were provided with three 100 ml bottles of lotion to last one month (approximately 10 ml per day). Children under 12 years were provided with two bottles per month, corresponding to approximately 7 ml per day. This amount was considered sufficient to apply the treatments to arms and legs as demonstrated by trial staff. Participants were instructed to use the lotion every evening. Full USA compliant consumer product information were given verbally in the local language. Any contraindications or side effects were recorded and reported at each monthly follow-up for appropriate action to local District Health departments.

Although previous repellent trials have used 20% DEET, a 15% DEET formulation was selected because this was the lowest concentration shown to be effective against mosquito biting in the study area [36]. A low concentration was desirable to minimise the possibility of adverse events in study subjects as the trial would require them to use the repellent for up to nine months.

All study households were provided with sufficient LLINs (PermaNet®2.0, deltamethrin 55 mg/m^2^, mesh 25 holes/cm^2^), defined as one net for every 1.5 persons in the household, plus another for use away from home. Participants were instructed to sleep under a net every night, particularly when away from the village. At monthly follow-up visits participants reported how many nights they had slept under the nets in the village and also when sleeping in the forest or rice fields.

Compliance was measured through self-reported use as nights per month and proportion of lotion used estimated from returned bottles. Random checks were carried out by trial staff to monitor compliance, which involved visiting a village at dusk and smelling the arms of participants to check lotion had been applied. To be included in the according to protocol analysis a participant was required to have used the lotion over 90% over the time: so self-reported lotion use should be more than 27 evenings per month and the volume of lotion used over 270 ml per month for adults and 180 ml for children.

The repellent and placebo lotions were identified by 3-digit codes as provided by the manufacturer. Participants, field staff carrying out randomisation and follow-up surveys and trial staff performing data entry and analysis were blinded for the length of the trial. The trial was double-blinded and unblinding was only carried out after data analysis was complete. However, the possibility remains that participants were able to distinguish between the active repellent and the placebo by the effect on biting insects.

### Follow-up surveys

All participants were tested by rapid diagnostic test (RDT: CareStart™ Malaria Pf/Pv Combo test, AccessBio, NJ) every month during active case detection. Follow-up surveys finished in December in both years to ensure testing throughout the wet season and into the following transition/dry season when previous surveys had found high parasite rates [19]. All positive cases were referred for immediate treatment following local guidelines through the district health teams working on the study.

The primary outcome was malaria incidence measured by monthly RDTs for *P. falciparum* and *P. vivax*. Positive RDTs, paired with a negative RDT matched by age and village, were confirmed by polymerase chain reaction at the London School of Hygiene & Tropical Medicine.

### Statistical analyses

The analytical plan was written prior to data collection. To avoid the risk of including relapse infections, the plan was to only include the first *P. vivax* positive result for each participant. However, in the event no participants presented with multiple *P. vivax* infections and all positive cases were therefore assumed to be new infections. The low malaria rates also meant that the original analysis using Poisson regression adjusted for repeated measures and household clustered was unnecessarily complicated and a simpler model chosen here.

An intention-to-treat analysis was performed on all data, followed by a per protocol analysis which excluded participants who slept under an LLIN and used the lotion in the evenings less than 90% of the month. Subsidiary analyses were also carried out at 75% and 50% compliance cut-offs. A principal component analysis (PCA) using data on education, house construction materials, type of electricity supply, ownership of motorbikes, tractors and televisions and animal ownership was carried out to establish overall socio-economic scores for each household. The PCA scores along with the age, gender, nights slept under an LLIN and nights spent away from the village were considered for inclusion in the regression. All variables except treatment group were first tested by non-parametric univariate methods and those with a significant association with the outcome at p<0.2 were considered for inclusion in the final model. Outcomes of time to first malaria, *P. falciparum* and *P. vivax* infection were tested by Mantel-Cox regression adjusted for intra-cluster household variation by robust methods. Variables maintaining their association in the multivariable model at p<0.05 were kept in the final model. All analyses were carried out using STATA version 12 (Statcorp, Texas, USA).

### Changes to trial design

Lower than expected malaria rates during the first year of the trial led to a revision of the sample size required. Therefore 1300 households were recruited in the second year rather than the previously planned 700. This increase meant the sideline serological work on arbovirus infections had to be dropped due to insufficient funding, and the trial focused solely on malaria infections the major vector borne disease in the area.

## Results

### Baseline data

The first round of recruitment from 25^th^ June to 4^th^ July 2009 enrolled 300 households to the trial and a further 1,297 households were recruited between 24^th^ April and 18^th^ May 2010. A total of 7,980 participants were initially recruited but 40 (0.5%) were excluded after the baseline survey as they were outside the 6–60 years age limit. Almost half of households (795, 49.8%) were randomised to the repellent arm and the remaining 802 allocated to the placebo lotion. Baseline household-level socioeconomic scores were derived from principle components analysis on data about the occupation and education of household heads, house construction materials, possessions and animal ownership. Baseline malaria rates were slightly higher in the placebo group, but not significantly so (Risk ratio: 0.82, 95% C.I. 0.5–1.37, p = 0.454). Men had a slightly higher rate of malaria, but this did not reach significance (parasite rate in females: 0.61%, in males: 0.83%, p = 0.263). Children aged 6–10 years had the highest infection rates although differences were again not significant. Overall, the households in the two treatment groups were very similar despite wide variation between households (Table 1), illustrating the success of the randomisation procedure.

**Table 1:** Baseline characteristics of the study participants and households.

		Repellent	Placebo
% Participants female		55.3%	54.9%
Median age of participants in years (IQR)		19 (11–35)	20 (11–35)
Ethnicity by household:	Lao	396 (12.1%)	396 (12.3%)
Katuic		712 (21.8%)	726 (22.5%)
Bahnaric-Khmer		2154 (66.0%)	2106 (65.2%)
Median household PCA1 score (IQR)		−0.23 (−1.14, 1.09)	−0.23 (−1.23, 1.24)
Median household PCA2 score (IQR)		−0.07 (−0.78, 0.66)	−0.07 (−0.89, 0.80)
Parasite rate (%)		0.68	0.83

Note: A Principal Components Analysis was used to combine social and economic data into two scores PCA1 and PCA2.

### Trial progress

**Figure 2:**
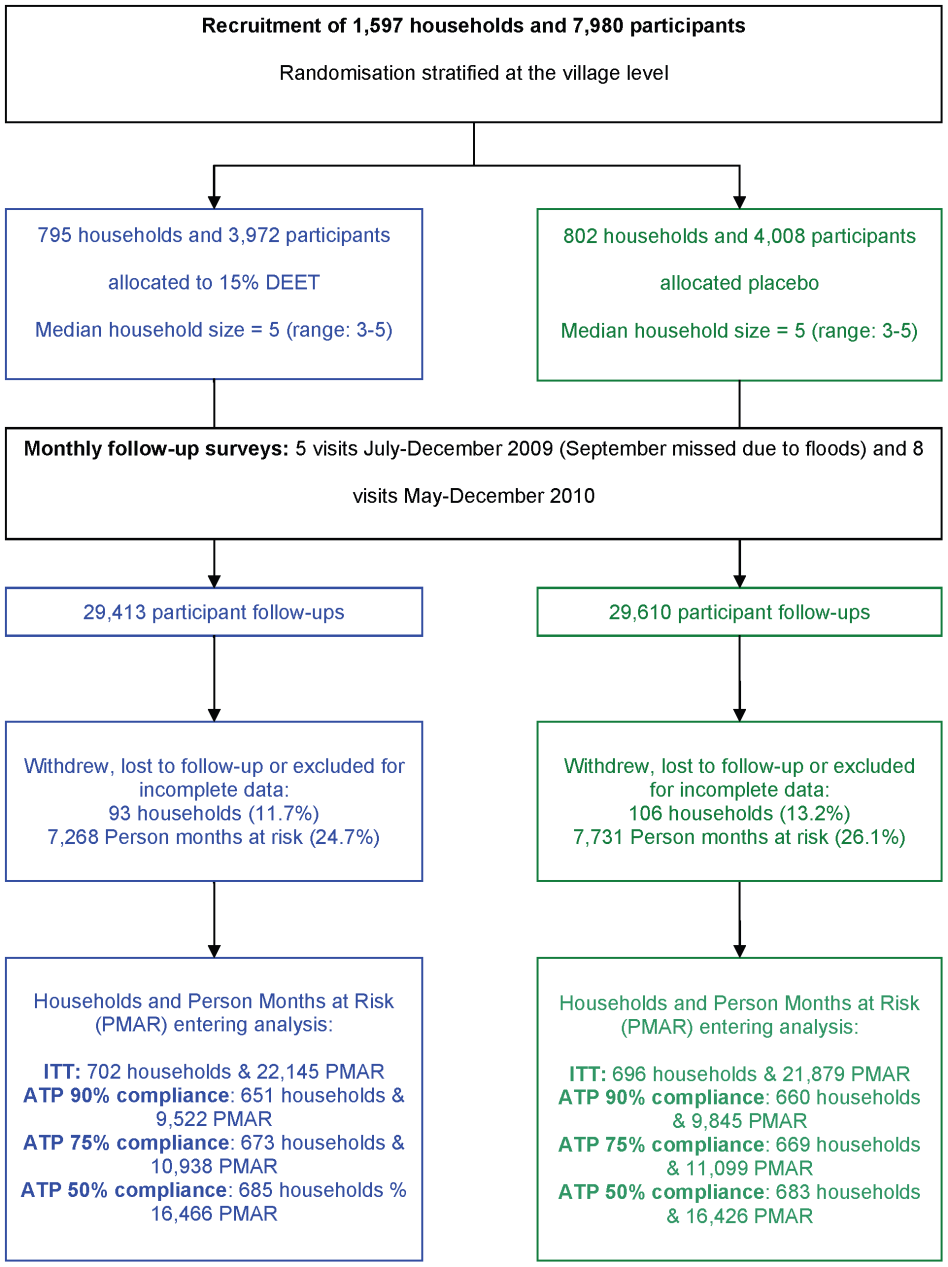
Progress of households from recruitment to Intention to Treat (ITT) and According to Protocol (ATP) analysis.

Follow-up visits were carried out monthly, finishing in December both years. However, no visits were made in September 2009 due to widespread flooding in the area, although households did receive monthly lotion supplies. 7,980 people were enrolled to the trial in June 2009 and April 2010. Forty participants were later found to be outside the 6–60 year age limit and excluded and a further 32 participants withdrew from the trial before follow-up. Thus 7,908 participants were followed up for a total of 4,218 person-years giving an average follow-up of 6.4 months per participant (Figure 2). Eighty-seven (1.1%) people experienced at least one malaria episode (Table 2).

**Table 2:** Malaria cases in repellent and placebo users.

	Repellent users	Placebo users
Participants	3,947	3,961
Mean follow-up (months)	6.4	6.4
Malaria	45	42
*P. falciparum*	35	33
*P. vivax*	14	16
Mixed infections	4	7

### Compliance

Approximately 60% of participants self-reported full compliance with lotion use each month with no difference between treatment arms (repellent users 61.3%, placebo users 62.2%, p = 0.104). Health staff also observed the volume of lotion that was returned and found less than half of participants had used all the lotion (repellent users 47.4%, placebo users 48.1%). A comparison of full compliance from these two measures showed the false positive rate, self-reported full compliance with non-lotion use was 46.7%, was much higher than the false negative rate, complete lotion use with self-reported non-compliance 28.5% (Table 3).

**Table 3:** Observed and self-reported lotion use per participant-month.

		Observed lotion use per month		
		<100%	100%	Total
Self-reported lotion use per month	<100%	14,122 (72.9%)	5,259 (27.1%)	19,381
	100%	12,339 (39.1%)	18,949 (60.6%)	31,288
Total		26,461 (52.2%)	24,208 (47.8%)	50,669

The most common reason for non-compliance in both repellent and placebo users was forgetting to use the lotion, although allowing for household clustering there was no significant difference between treatment arms (repellent users 68.8%, placebo users 69.1%, p = 0.675). Other reasons for no compliance included disliking the smell (repellent users 12.9%, placebo users 12.3%, p = 0.212), and allergies which were slightly higher in repellent users (repellent users 3.8%, placebo users 3.2%, p = 0.029).

Compliance with LLIN use was much higher than for lotion use regardless of treatment group and accounting for household clustering (repellent users 97.0%, placebo users 97.3%, p = 0.711). But a relationship was found between compliance with LLIN use and compliance with lotion use, and those participants who did not sleep under their LLIN every night were much less likely to use the lotion every day (χ^2^ = 316.1, p<0.001).

### Intention to treat analysis

Mean time to first malaria episode was 4.0 months (range: 0.9–7.5) in the placebo group and 3.9 months (range: 0.7–7.5) in the repellent group (Table 4). After accounting for socio-economic score and gender, a Mantel-Cox comparison of the hazard ratio found no difference between the two treatment groups (hazard ratio: 1.00, 95% CI 0.989–1.014, p = 0.868). Similarly, no differences were found between the two groups in terms of *P. falciparum* (hazard ratio: 1.00, 95% CI 0.989–1.02, p = 0.641) and *P. vivax* infection (hazard ratio: 1.00, 95% CI 0.979–1.02, p = 0.904).

**Table 4:** Mean time in person-months to first malaria infection in repellent and placebo users.

	Repellent users	Placebo users
Participants	45	42
Mean time to first malaria	3.9	4.0
Mean time to first *P. falciparum*	3.7	3.6
Mean time to first *P. vivax*	3.3	3.5
Mean time to first mixed infection	7.6	6.5

### According to protocol analysis

According to protocol analyses including only those participants who had both reported and observed compliance with lotion use above 50%, 75% and 90% also found no effect on malaria from the use of repellent (Table 5). A higher socio-economic score was found to significantly reduce malaria risk (hazard ratio: 0.72, 95% CI: 0.58–0.90, p = 0.004). Women were also found to have a reduced risk of infection (hazard ratio: 0.59, 95% CI: 0.37–0.92, p = 0.020).

**Table 5:** Risk ratios and significance of repellent use, gender and socio-economic score for malaria infection in Mantel-Cox regressions of participants who used lotion more than 50%, 75% and 90% of the time.

Model		50% Compliance	75% Compliance	90% Compliance
Malaria	Placebo	Comparison group	Comparison group	Comparison group
	Repellent	1.00 (p = 0.641)	1.00 (p = 0.730)	0.98 (p = 0.121)
	PCA1	0.82 (p = 0.117)	-	0.59 (p = 0.084)
	PCA2	-	1.31 (p = 0.039)	-
Falciparum	Placebo	Comparison group	Comparison group	Comparison group
	Repellent	0.99 (p = 0.406)	1.00 (p = 0.712)	0.99 (p = 0.498)
	Male	Comparison group	-	-
	Female	0.67 (p = 0.135)	-	-
	PCA1	-	-	0.59 (p = 0.062)
	PCA2	-	1.39 (p = 0.028)	

## Discussion

### Summary

A randomised placebo controlled trial of 15% DEET repellent lotion used by agricultural communities in southern Lao PDR was carried out over two wet seasons in 2009–2010. The trial was powered to detect an intervention effect of 50% on malaria incidence. No significant reduction in malaria incidence was found from the use of the topical repellent.

The regression analysis identified socio-economic scores as being the most important risk factors for malaria. This score was derived from data on household possessions and house building materials. An increase of 1 in this score could represent having a tiled roof compared to a thatched one, ownership of 1.5 more motorbikes or 1 more television and corresponded with a 20–45% reduction in the risk of malaria. Although malaria researchers in the Lao PDR have not previously looked for a link between malaria risk and wealth, our results are consistent with the findings of other studies where lower socio-economic status is associated with increased malaria risk [37], [38].

There was a sustained drop of over 50% in monthly malaria prevalence from baseline when all households were provided with LLINs. Although this effect could be a result of changes in malaria as there was no control group, the fact that lower prevalences continued to be recorded throughout the wet season when they would have been expected to increase supports the view that LLINs and repeated treatment of malaria patients was effective at reducing overall malaria transmission. However, the baseline rates in 2009 and 2010 were similar suggesting there had been no overall drop in malaria in the area between the two years.

### Limitations

The use of 15% DEET was chosen based on human landing catches in a village in rural Lao PDR [36]. This meant that the protection measured would accurately reflect the perception of biting pressure experienced by the participants of the trial. However the major malaria vectors in the area, *An. minimus* and *An. maculatus*, were very rare in the pilot study, so the level of protection by 15% DEET from these species was not tested. Anophelines show less response to repellents than other genera, including *Stegomyia* and *Culex* mosquitoes that made up the majority of catches in the local area [39], [40]. Therefore the recorded 98.9% protection against biting from 15% DEET is potentially an overestimate for the protection from malaria vectors.

While this gap in the efficacy testing of the intervention should be acknowledged, it is probably not as important as the variation in the dosage of DEET applied to the skin that would result from variation between user applications. A participant applying only 5 ml of the repellent lotion, would achieve the same DEET dosage as 10 ml of a 7.5% DEET lotion. Even two participants applying the same volume of lotion would end up with slightly different dosages depending on their relative body surface area. This variability is a major limitation with topical insect repellents as an intervention tool, but this does not rule out other forms of repellent such as impregnated fabrics that can be better standardised.

Our pilot study found a reduction in mosquito biting of over 95% when individuals used the trial repellent compared to the placebo, indicating repellent users might easily be able to distinguish which group they had been assigned to after a short period of use. All households in one treatment arm from a particular village withdrew from the trial after three months because they believed they had the placebo rather than repellent lotion, and unblinding found that they were correct. This meant that although the allocation of treatments and analysis was carried out blind, it is unlikely that all participants remained successfully blinded throughout the trial. An alternative that could prevent the two lotions being directly compared would be to assign separate batch numbers to each household's supply meaning there was no easy way for participants to separate the two treatment groups. This method was not implemented in this trial as distribution of lotion was made each month by village health workers with limited education and it was judged that the system should work as simply as possible to avoid confusion.

In order to reduce the possibility of artificially increasing malaria rates in the placebo group through diversion of biting from repellent users, a maximum of 25% of any village were recruited to the trial. However although all members of a household were randomised to the same study arm, it was not feasible to enforce repellent use by all members of a household at all times. Therefore, diversion of mosquitoes from participants using the repellent to participants within the same household not using the repellent could increase malaria risk for those individuals. Individual compliance could have a large impact on this household randomised trial, and the ability to accurately measure this could also have an important impact on the outcome. Participants were not compelled to use repellent during this trial, a demonstration of how much, where and when to apply the lotion was given at the start of the trial. Participants self-reported the number of evenings per month they used the lotion and as a second measure the amount of lotion returned was recorded. Random checks were also carried out in the evenings on a small sample of villages. Self-reported data on compliance is notoriously unreliable, so these data were verified by the volume of lotion used, as reported by interviewers, in order to filter non-compliers out of the ATP analysis. However uncertainty remained over the actual daily use of the lotion, in particular whether all members of a single household had used the lotion supplied equally.

A small number of RDT tests were verified by PCR and a single false negative was recorded. This raises the possibility that some infections were missed by the pLDH based tests used in this trial. Sub-microscopic infections have been shown to be of greater importance for malaria transmission in low-transmission settings such as were found in southern Lao PDR [41]. It is therefore recommended that future trials in this region should include PCR analysis to ensure non-falciparum infections are not missed.

Compliance was lower in this trial than in previous repellent trials. Self-reported and observed data gave estimates of full compliance from 48–60%, other trials have reported compliance levels from 68–98% [11], [14], [15]. This trial ran for a longer period (8 months compared to an average 4–6 months) which may have resulted in lower compliance, particularly in drier months with low mosquito numbers. However no decline in compliance was seen in this trial from month to month, and the length of the trial did not obviously correlate with the compliance in other trials. One trial did report compliance around 50%, but this was because not enough repellent had been provided [13]. Mathematical modelling suggests that compliance would be the most important influence on the success of repellent interventions, so this low level of compliance may explain at least some of the lack of effect found in our results [42].

This trial was focused on agricultural populations and the results may not be applicable to one important malaria risk group within the GMS. Forestry workers spend much more time in the forest potentially without access to good healthcare, and live in more temporary accommodation meaning they may be more exposed to vector biting. They often come from elsewhere in the region and their movement between endemic and non-endemic areas has been linked to the spread of antimalarial resistance in the region [16].

### Conclusion

Southern Lao PDR shares similarities in malaria vectors, environment and the human population with much of the GMS and the results of this trial are likely to be applicable across this region. Topical repellents are not likely to be a suitable intervention for agricultural populations in this region already using LLINs who require long-term protection throughout the wet season.

## Supporting Information

Protocol S1 **Can the insect repellent** ***N,N*** **-diethyl-** ***m*** **-toluamide (DEET) provide additional protection against clinical malaria over current best practice?** A cluster-randomised controlled trial.
(DOC)

Checklist S1 **CONSORT 2010 checklist of information to include when reporting a randomised trial.**
(DOC)

Analysis S1 **Poisson Regression Analysis.**
(DOC)

Analysis S2 **Principal Components Analysis.**
(DOC)

## References

[1] WHO (2011) World Malaria Report 2011. World Health Organization, Geneva.

[2] Sinka M, Rubio-Palis Y, Manguin S, Patil A, Temperley W, et al (2010) The dominant *Anopheles* vectors of human malaria in the Americas: occurence data, distribution maps and bionomic precis. Parasites & Vectors 3: 72. 20712879 10.1186/1756-3305-3-72 PMC2936890

[3] Sinka M, Bangs M, Manguin S, Chareonviriyaphap T, Patil A, et al (2011) The dominant *Anopheles* vectors of human malaria in the Asia-Pacific region: occurrence data, distribution maps and bionomic précis. Parasites & Vectors 4: 89. 21612587 10.1186/1756-3305-4-89 PMC3127851

[4] Trung H, Van Bortel W, Sochantha T, Keokenchanh K, Briet O, et al (2005) Behavioural heterogeneity of *Anopheles* species in ecologically different localities in Southeast Asia: a challenge for vector control. Tropical Medicine and International Health 10: 251–262. 15730510 10.1111/j.1365-3156.2004.01378.x

[5] Bayoh M, Mathias D, Odiere M, Mutuku F, Kamau L, et al (2010) *Anopheles gambiae*: historical population decline associated with regional distribution of insecticide-treated bed nets in western Nyanza Province, Kenya. Malaria Journal 9: 62. 20187956 10.1186/1475-2875-9-62 PMC2838909

[6] Russell T, Govella N, Azizi S, Drakeley C, Kachur S, et al (2011) Increased proportions of outdoor feeding among residual malaria vector populations following increased use of insecticide-treated nets in rural Tanzania. Malaria Journal 10: 80. 21477321 10.1186/1475-2875-10-80 PMC3084176

[7] Taylor B (1971) Changes in the feeding behaviour of a malaria vector, *Anopheles farauti* Lav., following use of DDT as a residual spray in houses in the British Solomon Islands Protectorate. Transactions of the Royal Entomological Society of London 127: 277–292.

[8] Yap H (1986) Effectiveness of soap formulations containing DEET and permethrin as personal protection against outdoor mosquitoes in Malaysia. Journal of the American Mosquito Control Association 2: 63–7. 2906963

[9] Yap H, Jahangir K, Zairi J (2000) Field efficacy of four insect repellent products against vector mosquitoes in a tropical environment. Journal of the American Mosquito Control Association 16: 241–4. 11081653

[10] Lindsay S, Ewald J, Samung Y, Apiwathnasorn C, Nosten F (1998) Thanaka (*Limonia acidissima*) and deet (di-methyl benzamide) mixture as a mosquito repellent for use by Karen women. Medical and Veterinary Entomology 12: 295–301. 9737602 10.1046/j.1365-2915.1998.00115.x

[11] McGready R, Simpson J, Htway M, White N, Nosten F, et al (2001) A double-blind randomized therapeutic trial of insect repellents for the prevention of malaria in pregnancy. Transactions of the Royal Society of Tropical Medicine and Hygiene 95: 137–8. 11355542 10.1016/s0035-9203(01)90137-3

[12] Rowland M, Freeman T, Downey G, Hadi A, Saeed M (2004) DEET mosquito repellent sold through social marketing provides personal protection against malaria in an area of all-night mosquito biting and partial coverage of insecticide-treated nets: a case-control study of effectiveness. Tropical Medicine and International Health 9: 343–50. 14996363 10.1046/j.1365-3156.2003.01183.x

[13] Kroeger A, Gerhardus A, Kruger G, Mancheno M, Pesse K (1997) The contribution of repellent soap to malaria control. American Journal of Tropical Medicine and Hygiene 56: 580–4. 9180612 10.4269/ajtmh.1997.56.580

[14] Rowland M, Downey G, Rab A, Freeman T, Mohammad N, et al (2004) DEET mosquito repellent provides personal protection against malaria: a household randomized trial in an Afghan refugee camp in Pakistan. Tropical Medicine and International Health 9: 335–42. 14996362 10.1111/j.1365-3156.2004.01198.x

[15] Hill N, Lenglet A, Arnez A, Carneiro I (2007) Plant based insect repellent and insecticide treated bed nets to protect against malaria in areas of early evening biting vectors: double blind randomised placebo controlled clinical trial in the Bolivian Amazon. British Medical Journal 335: 1023. 17940319 10.1136/bmj.39356.574641.55 PMC2078668

[16] WHO Mekong Malaria Programme (2010) Malaria in the Greater Mekong Subregion: Regional and country profiles. World Health Organization South-East Asia Region and WHO Western Pacific Region, Bangkok.

[17] WHO (2008) Country Health Information Profiles: Lao People's Democratic Republic. World Health Organization, Geneva.

[18] Shirayama Y, Phompida S, Kuroiwa C (2008) Monitoring malaria control in Khammouane province, Laos: an active case detection survey of *Plasmodium falciparum* malaria using the Paracheck rapid diagnostic test. Transactions of the Royal Society of Tropical Medicine and Hygiene 102: 743–50. 18589463 10.1016/j.trstmh.2008.05.014

[19] Vythilingam I, Sidavong B, Chan S, Phonemixay T, Vanisaveth V, et al (2005) Epidemiology of malaria in Attapeu Province, Lao PDR in relation to entomological parameters. Transactions of the Royal Society of Tropical Medicine and Hygiene 99: 833–9. 16112154 10.1016/j.trstmh.2005.06.012

[20] Phetsouvanh R, Vythilingam I, Sivadong B, Hakim S, Chan S, et al (2004) Endemic malaria in four villages in Attapeu Province, Lao PDR. Southeast Asian Journal of Tropical Medicine and Public Health 35: 547–51. 15689064

[21] Obsomer V, Defourny P, Coosemans M (2007) The *Anopheles dirus* complex: spatial distribution and environmental drivers. Malaria Journal 6: 26. 17341297 10.1186/1475-2875-6-26 PMC1838916

[22] Sidavong B, Vythilingam I, Phetsouvanh R, Chan S, Phonemixay T, et al (2004) Malaria transmission by *Anopheles dirus* in Attapeu Province, Lao PDR. Southeast Asian Journal of Tropical Medicine and Public Health 35: 309–15. 15691129

[23] Baimai V, Kijchalao U, Sawadwongporn P, Green C (1988) Geographic distribution and biting behaviour of four species of the *Anopheles dirus* complex (Diptera: Culicidae) in Thailand. Southeast Asian Journal of Tropical Medicine and Public Health 19: 151–161. 3406803

[24] Vythilingam I, Keokenchan K, Phommakot S, Nambanya S, Inthakone S (2001) Preliminary studies of *Anopheles* mosquitos in eight provinces in Lao PDR. Southeast Asian Journal of Tropical Medicine and Public Health 32: 83–7. 11485101

[25] Vythilingam I, Phetsouvanh R, Keokenchanh K, Yengmala V, Vanisaveth V, et al (2003) The prevalence of *Anopheles* (Diptera: Culicidae) mosquitoes in Sekong Province, Lao PDR in relation to malaria transmission. Tropical Medicine and International Health 8: 525–35. 12791058 10.1046/j.1365-3156.2003.01052.x

[26] Vythilingam I, Sidavong B, Thim C, Phonemixay T, Phompida S, et al (2006) Species composition of mosquitoes of Attapeu Province, Lao People's Democratic Republic. Journal of the American Mosquito Control Association 22: 140–3. 16646338 10.2987/8756-971X(2006)22[140:SCOMOA]2.0.CO;2

[27] UNDP (2001) National Human Development Report Lao PDR 2001. Advancing Human Development. United Nations Development Programme, Vientiane.

[28] Toma T, Miyagi I, Okazawa T, Kobayashi J, Saita S, et al (2002) Entomological surveys of malaria in Khammouane Province, Lao PDR, in 1999 and 2000. Southeast Asian Journal of Tropical Medicine and Public Health 33: 532–46. 12693588

[29] Socheath S, Seng C, Rath T, Deesin V, Deesin T, et al (2000) Study on bionomics of principal malaria vectors in Kratie Province, Cambodia. Southeast Asian Journal of Tropical Medicine and Public Health 31: 106–110. 11414438

[30] Trung H, Van Bortel W, Sochantha T, Keokenchanh K, Quang N, et al (2004) Malaria transmission and major malaria vectors in different geographical areas of Southeast Asia. Tropical Medicine and International Health 9: 230–237. 15040560 10.1046/j.1365-3156.2003.01179.x

[31] Tun-Lin W, Thu M, Than S, Mya M (1995) Hyperendemic malaria in a forested, hilly Myanmar village. Journal of the American Mosquito Control Association 11: 401–407. 8825497

[32] Socheat D, Denis M, Fandeur T, Zhang Z, Yang H, et al (2003) Mekong malaria. II. Update of malaria, multi-drug resistance and economic development in the Mekong region of Southeast Asia. Southeast Asian Journal of Tropical Medicine and Public Health 34: 1–102. 15906747

[33] Moore S, Davies C, Hill N, Cameron M (2007) Are mosquitoes diverted from repellent-using individuals to non-users? Results of a field study in Bolivia. Tropical Medicine and International Health 12: 532–9. 17445144 10.1111/j.1365-3156.2006.01811.x

[34] PSI Research Division (2008) Personal Communication.

[35] Hayes R, Bennett S (1999) Simple sample size calculation for cluster-randomized trials. International Journal of Epidemiology 28: 319–326. 10342698 10.1093/ije/28.2.319

[36] Chen-Hussey V (2012) A cluster-randomised trial to assess whether the insect repellent N,N-diethyl-m-toluamide (DEET) can provide additional protection against clinical malaria over current best practice in Lao PDR. Thesis: London School of Hygiene & Tropical Medicine.

[37] Bates I, Fenton C, Gruber J, Lalloo D, Medina L, et al (2004) Vulnerability to malaria, tuberculosis, and HIV/AIDS infection and disease. Part 1: determinants operating at individual and household level. Lancet Infectious Diseases 4: 267–77. 15120343 10.1016/S1473-3099(04)01002-3

[38] Worrall E, Basu S, Hanson K (2005) Is malaria a disease of poverty? A review of the literature. Tropical Medicine and International Health 10: 1047–59. 16185240 10.1111/j.1365-3156.2005.01476.x

[39] Barnard D (1998) Mediation of deet repellency in mosquitoes (Diptera: Culicidae) by species, age, and parity. Journal of Medical Entomology 35: 340–3. 9615557 10.1093/jmedent/35.3.340

[40] Curtis CF, Lines JD, Ijumba J, Callaghan A, Hill N, et al (1987) The relative efficacy of repellents against mosquito vectors of disease. Medical and Veterinary Entomology 1: 109–19. 2908762 10.1111/j.1365-2915.1987.tb00331.x

[41] Okell L, Bousema T, Griffin J, Ouédraogo A, Ghani A, et al (2012) Factors determining the occurrence of submicroscopic malaria infections and their relevance for control. Nature Communications 3 10.1038/ncomms2241 PMC3535331 23212366

[42] Kiszewski A, Darling S (2010) Estimating a mosquito repellent's potential to reduce malaria in communities. Journal of Vector Borne Diseases 47: 217–221. 21178214

