# Supplementary material for: Can Topical Insect Repellents Reduce Malaria? A Cluster-Randomised Controlled Trial of the Insect Repellent N,N-diethyl-m-toluamide (DEET) in Lao PDR
Source: PLoS One. 2013 Aug 14;8(8):e70664. doi: 10.1371/journal.pone.0070664 (PMC3743820; doi:10.1371/journal.pone.0070664)
Supplement: Protocol S1 — Can the insect repellent N,N -diethyl- m -toluamide (DEET) provide additional protection against clinical malaria over current best practice? A cluster-randomised controlled trial. (DOC) [file pone.0070664.s001.doc]

# PROTOCOL S1: CAN THE INSECT REPELLENT *N, N-*DIETHYL-*M*-TOLUAMIDE (DEET) PROVIDE ADDITIONAL PROTECTION AGAINST CLINICAL MALARIA OVER CURRENT BEST PRACTICE? A CLUSTER-RANDOMISED CONTROLLED TRIAL

## KEY ROLES

**Principal Investigator:** Professor Steve Lindsay, Disease Control & Vector Biology Unit, London School of Hygiene and Tropical Medicine, Keppel Street, London WC1e 7HT, UK, Steve.Lindsay@lshtm.ac.uk

**Investigator:** Vanessa Chen-Hussey, Disease Control & Vector Biology Unit, London School of Hygiene and Tropical Medicine, Keppel Street, London WC1e 7HT, UK

**Statistician and Epidemiologist**: Ilona Carniero, Disease Control & Vector Biology Unit, London School of Hygiene and Tropical Medicine, Keppel Street, London WC1e 7HT, UK

**Other Institutions:** Rob Gray, Country Director, Population Services International (PSI) Laos, Saphanthong Tai, Sisattanak District, Vientiane, Lao PDR

## ABBREVIATIONS

DALY: Disability Adjusted Life Years

DEET: Di-ethyl-*M*-toluamide

IgM: Immunoglutinin M

IgG: Immunoglutinin G

IRS: Indoor Residual Spraying

ITN: Insecticide Treated Net

JE: Japanese Encephalitis

Lao PDR: Lao People’s Democratic Republic

LLIN: Long Lasting Insecticide Treated Net

LSHTM: London School of Hygiene & Tropical Medicine

NOEL: No Observed Effect Level

PMD: *p*-Methane-3,8-diol (insect repellent from lemon eucalyptus)

PSI: Population Services International (social marketing organisation)

RDT: Rapid Diagnostic Test

*s.l.*: *sensu lato*

*s.s.*: *sensu stricto*

VHV: Village Health Volunteer

WHO: World Health Organization

## PROTOCOL SUMMARY

Population

Approximately 8,000 rural agricultural workers and children aged from 6 to 60 years, resident in 126 villages in Sekong and Attapeu Provinces in southern Lao PDR.

Sites

One

Study Duration

March 2009 to Feb 2012

Subject Participation Duration

Either 7 months from June to December 2009, or 9 months from April to December 2010

Interventions

1. A single Long-lasting insecticide treated net (PermaNet®2.0, deltamethrin 55mg/m^2^, mesh 25 holes/cm2) for every 1.5 people in the household

2. 15% DEET lotion or a placebo lotion (both supplied by SCJohnson)

Objectives: The aim of the study is to evaluate the efficacy of 15% DEET insect repellent to reduce malaria incidence in rural populations living in southern Lao PDR.

Study Design: The study will take place in Attapeu and Sekong Provinces in southern Lao PDR and will involve 8000 subjects clustered by household. Participants will be primarily recruited amongst rural agricultural populations who often work and sleep away from the village during the wet season. Either a DEET or placebo lotion will be provided for participants to use every evening. All participants will be supplied with long lasting insecticide treated nets (LLINs). Data collection will take place during June to December of 2009 and April to December of 2010. Malaria cases will be identified through active case detection using RDTs (rapid diagnostic tests) at baseline and at monthly intervals post-intervention. Secondary outcomes are all-cause fevers self reported via monthly questionnaires, and dengue, JE and typhus IgM/IgG from bloodspots collected at baseline and exit.

ClinicalTrials.gov Identifier: NCT00938379

## BACKGROUND

### Study Area

Attapeu and Sekong Provinces in south-eastern Lao PDR border both Cambodia to the south and Vietnam to the west. The wet season runs from April to October, followed by a cool dry season November to January and a hot dry season February to March. Rice farming is the main economic activity in these two provinces, 57% and 71% of Attapeu and Sekong’s populations are farmers respectively (UNDP, 2001).

### Malaria in Lao PDR

Malaria is a major public health problem in Lao PDR: the World Health Organization estimates that malaria was responsible for 46 lost DALYs per 100,000 population in 2004 (WHO, 2009). The prevalence of malaria recorded in Lao PDR in 1998 was 55 cases per 1,000 population; but in Attapeu the prevalence was 101 per 1,000 and in Sekong it was 163 per 1,000 the highest rate in the country (UNDP, 2001).

Current policy is for the entire population at risk (estimated to be 70% of the country) to receive insecticide treated nets (WHO, 2005). In addition free diagnosis and ACT (artemisinin combined therapy) treatment has been implemented to poor populations. Artemisine resistance has been recorded in neighbouring Cambodia, although it is not yet recorded in Laos (Singhasivanon *et al.* 2003).

In general the use of DEET (*N,N-*diethyl-*M*-toluamide) repellents by rural people is often low due to cost, but other methods of personal protection such as coils or repellents made from locally available ingredients such as neem oil are still used (Frances and Wirtz, 2005).

### Malaria Vector Behaviour in Lao PDR

The main malaria vectors in Lao PDR are *Anopheles dirus*, *An. minimus*, *An. maculatus* and *An. jeyporiensis* (Toma *et al.* 2002; Vythilingam *et al.* 2003; Vythilingam *et al.* 2005). Feeding time amongst the *An. dirus* complex is observed to vary with sibling species (Baimai *et al.* 1988), in Laos the highest biting frequencies are recorded between 21.00h and 02.00h (Toma *et al.* 2002; Vythilingam *et al.* 2003; Vythilingam *et al.* 2005). No preference for indoor or outdoor feeding has been observed for *An. dirus s.l.* in Lao PDR (Vythilingam *et al.* 2003; Vythilingam *et al.* 2005). An early feeding cycle has been found for *An. minimus* *s.l.* with most biting occurring from 18.00h to midnight (Tun-Lin *et al.* 1995; Rattanarithikul *et al.* 1996; Zhou 2003). Several studies have also found a second peak following this around 01.00h (Chareonviriyaphap *et al.* 2003; Vythilingam *et al.* 2003; Sungvornyothin *et al.* 2006). Most studies have recorded little or no indoor or outdoor feeding preference in *An. minimus s.l.* (Socheath *et al.* 2000; Chareonviriyaphap *et al.* 2003; Tun-Lin *et al.* 2003; Vythilingam *et al.* 2003; Trung *et al.* 2005; Vythilingam *et al.* 2005; Sungvornyothin *et al.* 2006) although a comparison of sibling species found *An. harrisoni* to be more exophagic than *An. minimus s.s.* (Van Bortel *et al.* 1999). Strongly exophagic behaviour is recorded for *An. maculatus s.l.* throughout Southeast Asia (Schultz 1992; Torres *et al.* 1997; Socheath *et al.* 2000; Hassan *et al.* 2001; Vythilingam *et al.* 2003; Trung *et al.* 2005). It also generally feeds early from 18.00h to around 22.00h (Schultz 1992; Rattanarithikul *et al.* 1996; Socheath *et al.* 2000; Vythilingam *et al.* 2003; Zhou 2003). *An. jeyporiensis* shows a high level of behavioural plasticity; recorded feeding predominately indoors in Lao PDR, but mostly outdoors in Vietnam (Vythilingam *et al.* 2003; Trung *et al.* 2005). This is possibly due to the presence of sibling species, or misidentification as *An. jeyporiensis* is very difficult to distinguish morphologically from *An. harrisoni* (Do *et al.* 2008). Similarly, peak biting is recorded between 23.00h and 03.00h in Myanmar (Oo 2003), but from 22.00h to 06.00h in Lao PDR (Vythilingam *et al.* 2003). As biting can start as early as 18.00h and often takes place outdoors, bednets may not provide complete protection from malaria transmission

### Repellent Protection from Biting

Repellent trials have tested concentrations of DEET from 5-75%, in the laboratory and in the field (Table 1), however different mosquito densities and methodologies make comparisons difficult. One particular problem is the proximity of controls and treatments, which if too close can result in diversion of mosquitoes from treated to control, artificially distorting the difference between the catches (Moore *et al.* 2007). However, concentrations of DEET below 15% do not appear to give useful levels of protection.

### Table 1. Design and outcomes of DEET repellency trials against Southeast Asian anophelines

| Trial design | Outcome |
| --- | --- |
| Untreated arm was placed in a cage with 200 *An. dirus* for 1 minute. Then the arm was treated with 5%, 10% or 20% DEET and placed in the cage for 5 minutes. The treated arm was placed in the cage for 5 minutes every 30 minutes following until three bites were recorded, giving the protection time (Frances *et al.* 1996a). | Average protection time was less than 5 minutes for 5% DEET, 7.5 minutes for 10% DEET and 105.5 minutes for 20% DEET. |
| 25% DEET or 100% ethanol control applied to lower legs 1 hour before the start of collections. Outdoor landing catches performed in rubber tree plantation in Thailand for 7 hours starting at 18.00h or 19.00h. *An. dirus* was most common species (Frances *et al.* 1996a) | 25% DEET gave over 80% protection for up to 4 hours, and 50-70% protection for hours 5-7 following application. |
| 33%, 50% and 75% DEET applied to exposed skin and outdoor landing catches performed in forest area in Thailand from 18.00h to midnight. Controls were untreated. *An. dirus* made up 35% of catch, but density was very low (Frances *et al.*  1996b). | 33% DEET gave complete protection for 8 hours as did 75% DEET, although the 50% formulation only protected for 6 hours. |
| 20% DEET and 0.5% permethrin trialled in three different formulations against two controls (70% ethanol and thanaka and water). One treatment applied to arms and legs and pairs with same treatment spaced at least 10m apart. Anophelines in order of biting density; *An. maculatus*, *An. minimus* and *An. dirus*. Landing catches were carried out both indoors and outdoors from 18.00h to midnight, then indoors only until 06.00h, with additional catches outdoors from 05.00h to 07.00h (Lindsay *et al.* 1998). | 98.2-99.6% protection against all mosquitoes. |
| Outdoor catches from 21.00h to 01.00h in non-malarial rural residential area in Malaysia where *Anopheles sinensis* is most abundant species biting humans. 15% DEET in ethanol applied to one side of the body and 12% Bayrepel applied to other, while controls 5m away were untreated (Yap *et al.*  2000). | 15% DEET gave complete protection for 1 hour and 97.5% protection over 8 hours. |
| An arm treated with 20% DEET placed into a cage of 250 *An. dirus* for three minutes every 30 minutes for 12 hours between 18.00h and 06.00h. Protection time defined as time between application and either two bites in a three minute observation period or two consecutive observations with one bite (Thavara *et al.* 2001). | Protection time for 20% DEET against *An. dirus* was 5.8 hours. |
| Volunteers were treated with 20% DEET on one leg and 20% IR3535 on the other. Untreated controls were 1m away. Outdoor landing catches were carried out from 19.00h to 24.00h in four sites across Thailand (Thavara *et al.* 2001). | 20% DEET gave 94-100% protection from *Anopheles* biting for 5 hours. |
| 20% DEET and 0.5% permethrin soap applied to all exposed skin and outdoor landing catches carried out from 19.00h to 05.00h. Anopheline species included *An. nigerrimus* and *An. stephensi* (Rowland *et al.* 2004a). | 100% repellency for 10 hours from both culicine and anopheline biting. |

### Repellent Protection from Malaria

Repellent protection from biting is better demonstrated than protection from vector-borne disease transmission. Challenges to disease targeted repellent trials include the varying effect of repellents on different vector species (or even sibling species) and the acceptability of the repellent formulation used. Some of the earliest trials involved military personnel deployed to endemic areas in the 1940s which showed reductions in scrub typhus and sand fly fever incidence (Gupta and Rutledge, 1994).

In areas of seasonal malaria transmission in Ecuador and Peru, repellent soap (20% DEET and 0.5% permethrin) was distributed to nine communities over four months. *Plasmodium falciparum* was found in 86% of cases in Ecuador, while the predominant species in Peru was *P. vivax*. Another nine communities, paired for population, malaria incidence and access to health care, were controls. At the end of the four months, participants were interviewed to determine if they had experienced malaria during the trial. No significant difference was seen in malaria infection in either the control or intervention groups (Kroeger *et al.* 1997). It was suggested that the lack of effect could have been due to low uptake of the soap, it was estimated that the coverage of continuous soap use was only 50-70%. In addition, field trials showed that activity reduced the efficacy of the repellent formulation. It is also possible that the local vector, *An. albimanus*, is less susceptible to DEET than other anophelines.

Almost 900 pregnant women in a camp for displaced Karen people in Thailand were enrolled into a double-blind repellent trial. They were randomised to either 20% DEET in thanaka (a local plant-based cosmetic) or thanaka. The repellent or placebo was applied every evening for an average of 18 weeks per woman. A 28% reduction in *P. falciparum* was recorded in the intervention group, but this was not significantly different from the 15% reduction in the control group (McGready *et al.* 2001a).

In South Africa 15% DEET was applied twice an evening to ankles and feet of all 850 residents of a small community during a malaria outbreak following a flood. Malaria declined to pre-outbreak levels within four weeks, however without a control group in this case, the contribution of repellent to this decline is impossible to quantify (Durrheim and Govere 2002).

Following social marketing of a repellent soap (20% DEET and 0.5% permethrin) in Afghanistan, a case controlled study was carried out using malaria cases from local clinics. Controls were fever patients at the same clinics who tested negative for malaria. Participants were interviewed about their use of the repellent soap the day before, ten days before and for confounding factors such as ITN use. 96% of infections were from *P. vivax*. The use of repellent 10 days before a fever episode was associated with a 45% reduction in the odds of malaria, although this was not significant possibly due to low sample sizes (Rowland *et al.* 2004a).

The same repellent soap (20% DEET and 0.5% permethrin) was given to 67 households in an Afghan refugee camp in Pakistan. A similar number of households were provided with a placebo lotion. Repellent and placebo use appeared to be high, although this was only measured by a single round of interviews with 20 households. Passive case detection for six months found a 44% reduction in the odds of *P. falciparum* infection, although no effect was found for *P. vivax* (Rowland *et al.* 2004b).

A cluster randomised trial in Bolivia randomised 860 households to either 30% PMD (a repellent derived from lemon eucalyptus, *Eucalyptus maculata citriodon*) or 0.1% clove oil as a control. All participants were also provided with insecticide treated bed nets. *P. falciparum* infections were actively recorded by monthly rapid diagnostic tests, and *P. vivax* episodes were passively detected at local clinics. A per protocol analysis which excluded those who had not used the lotions for more than 10% of the time found and 80% reduction in *P. vivax* incidence in the treatment group. A reduction of 84% in *P. falciparum* incidence was also recorded in the treatment group, however this was not significant as case number were low (Hill *et al.* 2007).

### Study Rationale

Southeast Asian malaria vectors are known to bite outdoors in the evening as well as indoors during the night, meaning insecticide treated bed nets may only provide partial protection in these areas. It is hoped that repellents used during the evening could provide additional protection from malaria transmission over that given by treated bed nets. Following the encouraging results of a similar trial in Bolivia, this trial aims to establish whether Asian malaria vectors can also be prevented from transmitting malaria by the use of insect repellent.

### Potential Risks and Benefits

Finger-prick blood spots do cause modest, brief discomfort but are common in use in this area and it is likely most individuals will have already experienced this. Risk from this procedure is very low and antiseptic wipes are used to prevent infection.

Use of repellent is universal worldwide and present extremely low risk. We are using a high quality commercial brand made, registered and sold in the USA by SC Johnson. We are using the lowest effective dose of active 15% DEET and a consumer friendly gentle aqueous lotion formulation to minimise risk and discomfort. As young children have a lower body volume / skin surface ratio we will not enrol anyone under six years old. Full USA compliant consumer product information will be given verbally and in local language on the products. Any contraindications or side effects will be recorded and reported for appropriate action to local District Health departments. All positive cases will be referred for immediate treatment following local guidelines through the District Health teams working with us on the study. Treatment for *P. falciparum* with co-artem is free at the point of delivery in Lao PDR.

If findings are positive there would be a strong case for the use of repellents as part of future vector-borne disease control programmes.

## STUDY OBJECTIVES

### Objectives

The primary objective is to determine whether 15% DEET repellent used in the evening can reduce malaria incidence. Secondary objectives are to determine whether the intervention has an effect on fevers and dengue, Japanese Encephalitis (JE) and scrub typhus seroconversion. The trial will also monitor lotion acceptability and any occurrence of adverse events.

### Outcomes

The primary outcome measure will be the incidence of malaria defined as a positive rapid diagnostic test (RDT) result. Secondary outcomes will be all-cause fevers self reported via monthly questionnaires and seroconversion of dengue, JE and scrub typhus measured by ELISA test on dried bloodspots taken during baseline and exit surveys.

## METHODS

### Type of Study

A double-blind, randomised placebo-controlled trial of DEET repellent lotion used to prevent malaria transmission.

### Randomisation

Randomisation to repellent or placebo lotion will take place at the household level and is stratified by village. All the households in a village will be assigned to either group 258 or 305 (manufacturer codes of repellent and placebo lotions) during the baseline survey. District health workers who recruited and carried out the baseline survey labelled straws with the group codes which the heads of households then picked.

### Blinding

The repellent and lotion were labelled by the manufacturers with a 3-digit code, and will reveal which is which at the end of trial. The trial staff and local health staff carrying out randomisation and surveys are therefore blinded to the treatment arms. Participants were informed as part of the recruitment process that they had an equal chance of receiving the placebo or repellent. The possibility does remain of participants being able to distinguish the DEET lotion from the placebo by a slight difference in smell.

### Interventions

All participants will be provided with and sleep under long-lasting insecticide treated nets (LLINs) every night (PermaNet®2.0, deltamethrin 55mg/m^2^, mesh 25 holes/cm^2^). A 15% DEET lotion will be provided to half the households the other half of households will receive a placebo lotion (both supplied by SCJohnson). Adult participants will be provided with three 50ml bottles of their lotion to last one month (approximately 5ml per day). Children under 12 years will be provided with two bottles per month, corresponding to approximately 3ml per day. These exposures, based on Antwi *et al.* (2008), would have margins of exposure greater than 100 (exposure is less than 1% of NOEL) for acute and chronic toxicity. District health staff will demonstrate the amount of lotion to apply to arms and legs during the recruitment process. Participants are instructed to use the lotion every evening, but are free to also use the lotion during the day if desired.

## PARTICIPANT AND HOUSEHOLD ELIGIBILITY

Participants will be on primarily recruited amongst rural agricultural workers that often work and sleep overnight away from the village during the wet season. Households must enrol at least five eligible participants to enter the trial. Participants must be aged 6-60 years to take part in the trial.

## STUDY PROCEDURES AND EVALUATIONS

### Study Schedule

| Activity | 2009 | 2010 |
| --- | --- | --- |
| Design of consent form and survey forms | March | - |
| Design of database | March | - |
| Recruitment and training of Trial Coordinator | April | - |
| Design and approval of bi-lingual labels for lotions | April | - |
| Study design and SOPs approved by PSI and LSHTM | April | - |
| Translation of survey forms | April and May | - |
| Import and delivery of lotions, LLINs and RDTs | April-June | March-April |
| Gain ethics approval from LSHTM and Lao Ministry of Health | May | - |
| Training of District Health Staff | May-July & December | March-May & December |
| Selection of study villages | June | March |
| Training of VHVs | June, July & December | April, May & December |
| Recruitment and Baseline Surveys | June | April |
| Monthly follow-up Surveys | July-November | May-November |
| Evening spot checks | July-November | May-November |
| Entomology survey | July | July |
| Exit Survey | December | December |

### Recruitment and Baseline Survey

Recruitment will take place in May 2009, when 300 households will be recruited from Attapeu province, and in April 2010, when 1,300 households will be recruited from both Attapeu and Sekong provinces. Participants will be recruited as volunteers from meetings with village members and local health team staff where the study will be described in full with opportunity for questions. Individuals that wish to take part will also receive this information in writing and sign a consent form to confirm they understand the trial, including the possibility they may receive the placebo lotion. A maximum of 25% of households will be recruited from any village in order to overcome diversion of bites to non-repellent users. In addition district health staff will be responsible for ensuring that study houses contain at least five eligible participants (if the household is bigger, more can be recruited), and that study houses are distributed throughout the village, the rule being that study houses should be a minimum of 10m apart. The baseline survey is scheduled for the same time as recruitment, May 2009 and April 2010. The questionnaire, estimated to take 30 minutes, will collect basic information on wealth indicators and current malaria prevention behaviour for the household and individual information on malaria exposure.

Households will be randomly assigned to one of the treatment groups and supplied with three 100ml bottles per person per month to allow for 10ml use per day as well as sufficient LLINs for the whole household, including non-participants (sufficient LLINs defined as one net for every 1.5 persons in the household, plus another for use away from home). District health staff will demonstrate how to use the lotion and explain that it should be used every evening. Participants will also be encouraged to use the lotion during the day if they notice biting.

A bloodspot will also be taken from each participant and stored on filter paper for laboratory analysis for dengue, JE and typhus. Analysis will ideally take place in Laos if facilities can be found, but if this is not possible the bloodspots will be transported to the UK or France for testing.

### Follow-up Surveys

Monthly surveys will be carried out at monthly intervals after baseline and finishing in December in both years. The questionnaire will collect data on compliance, acceptability of the lotion and malaria exposure that month. An RDT will also be carried out to actively detect malaria cases. Positive results will be confirmed by PCR in London where possible and a random selection of negative RDTs will also be confirmed in this way. Positive cases are to be given prompt treatment according to local guidelines.

The access to many villages is anticipated to become very difficult during the height of the rainy season and the study aims to continue data collection by using village health volunteers (VHVs). These volunteers have already been trained in the use of RDTs and malaria treatment and will be further trained to give out replacement repellent/placebo and complete the monthly survey forms.

Access to remote communities is anticipated to be a major obstacle in data collection, particularly during the rainy season. The use of VHVs will hopefully enable data collection to continue throughout these months. The monthly survey has been designed to be as simple as possible to enable these workers who sometimes have limited education to complete the form.

### Exit Survey

An exit survey will be carried out during the final month of data collection, which will assess the acceptability of the repellent lotion in more detail. Another blood spot will be collected from each participant.

## SAFETY CONSIDERATIONS

### DEET Safety

DEET was first registered for use as an insect repellent over 50 years ago; it is effective against a wide range of insects and still remains the most widely used repellent today, with an estimated 200 million annual applications (Barnard 2000). However, DEET has been linked to adverse events following high exposures, primarily involving seizures and encephalopathy in children (Osimitz and Grothaus 1995). Qiu *et al.* (1998) reviewed the safety of DEET and found the compound associated with cases of encephalopathy, seizures and dermatitis; however concluded that DEET was safe when used appropriately given that the incidence of adverse effects was very low when its widespread use was considered.

About 5-8% of applied DEET is absorbed into the skin, but is subsequently completely metabolised and mostly excreted in the urine within four hours (Selim *et al.* 1995). Veltri *et al.* (1994) reviewed over 9,000 calls to American Poison Control Centres involving DEET exposure. Over half of exposures had no symptoms at the time of the call, and symptoms usually occurred through inhalation or contact with eyes. Almost 90% of exposures were treated solely at home, and 80% of those referred to a health centre were discharged after initial examination, suggesting mild or short-lived symptoms. Severity of symptoms was more closely related to the type of exposure than the concentration of DEET, or the age or gender of the patient. The United States Environmental Protection Agency reviewed DEET safety and concluded that it was not possible to identify DEET as the cause of seizures or encephalopathy and that there was no unreasonable risk to human health when used according to product instructions (USEPA 1998).

The safety of DEET use in pregnant women was assessed during a clinical trial of the product for the prevention of malaria (McGready *et al.* 2001b). No adverse neurological, gastrointestinal or dermatological effects were reported and neither were there any adverse effects on survival or development of the babies at birth or one year. Antwi *et al.* (2008) carried out a risk assessment of DEET use by humans. Based on animal tests a no-observed-effect-level (NOEL) was set at 200 mg DEET per kg body weight for acute toxicity, and 500 mg DEET per kg body weight dose per day was set for chronic toxicity.

## DISCONTINUATION CRITERIA

### Participant and Household Withdrawal

Participants may withdraw their consent to take part, as well as permission for their children to take part at any point before the end of the trial. If withdrawal takes place in the first two months of the trial, then further recruitment within the same village will be considered.

### Study Termination

The PI can decide to terminate the trial early if there is concern that the interventions are detrimental to participants’ health.

## STATISTICAL CONSIDERATIONS

### Sample Size Calculation

An initial target of 1,000 households was based on the power calculation below (Table 3). The only reliable clinical data from the study area at the time was 32% prevalence of *P. falciparum* from village surveys during September. The incidence over 9 months sampling was estimated to be 2-6%. Hill *et al.* [23] found 80% reduction in cases in their repellent group and Rowland *et al.* (2004b) found a 44% reduction in *P. falciparum*; therefore a 30-50% reduction was used in the calculations.

Sample sizes were recalculated following the first year’s data collection which found a 0.7% incidence of malaria. A sample size of 633 households per arm would therefore be necessary to detect a 50% intervention effect with 95% significance and 80% power. Non-compliance in the first year was around 27%, so the sample size was adjusted to 804 households per arm.

### Table 3. Initial sample size calculations

| Assumed incidence | Intervention effect | Coefficient of variation (K) | Power | Number of households in each arm |
| --- | --- | --- | --- | --- |
| 0.06 | 50% | 0.25 | 90% | 214 |
| 0.05 | 50% | 0.25 | 90% | 256 |
| 0.04 | 50% | 0.25 | 90% | 319 |
| 0.03 | 50% | 0.25 | 90% | 424 |
| 0.02 | 50% | 0.25 | 90% | 634 |
| 0.02 | 50% | 0.25 | 80% | 476 |
| 0.03 | 50% | 0.30 | 90% | 426 |
| 0.02 | 50% | 0.30 | 80% | 477 |
| 0.03 | 40% | 0.25 | 80% | 530 |
| 0.06 | 30% | 0.25 | 80% | 503 |

### Table 4. Revised sample size calculation following first year of data collection (giving a firmer estimate of malaria incidence), calculations are for different scenarios of effect with 95% significance and 80% power

| Assumed Incidence | Intervention Effect | Coefficient of variation (K) | Households Per Arm | Increase 27%  (Loss From Non-Compliance) |
| --- | --- | --- | --- | --- |
| 0.010 | 50% | 0.25 | 948 | 1204 |
| 0.015 | 50% | 0.25 | 633 | 804 |
| 0.020 | 50% | 0.25 | 476 | 605 |
| 0.010 | 40% | 0.25 | 1579 | 2006 |
| 0.015 | 40% | 0.25 | 1054 | 1336 |
| 0.020 | 40% | 0.25 | 792 | 1006 |
| 0.010 | 30% | 0.25 | 2982 | 3787 |
| 0.015 | 30% | 0.25 | 1991 | 2529 |
| 0.020 | 30% | 0.25 | 1495 | 1899 |

## DATA HANDLING AND RECORD KEEPING

### Data Analyses and Handling

Only the consent forms will contain personal information (names) and these will be stored for the length of the trial in a locked filing cabinet in the project office in Vientiane. Number codes will be used to identify participants during baseline and monthly surveys. Data will be entered into a password protected Microsoft Access database.

The initial analysis will be performed according to intention-to-treat. This will include all participants who were recruited to the trial and gave every monthly RDT result. An additional per protocol analysis will be carried out. This will include only those participants who slept under a bed net and used the lotion in the evenings at least 90% of the time. Positive cases will be subsequently excluded from the trial due to the possibility of them testing positive in later months from the same episode.

Data from both arms from baseline surveys will be compared by ANOVA. Comparison of *P. falciparum*, *P. vivax* and fever incidence between the trial arms will be carried out by poisson regression adjusted for intracluster (household) variation by random effects methods. Comparison of seroconversion for dengue, Japanese Encephalitis and scrub typhus between trial arms will be done using a chi-squared test on numbers seroconverted or not seroconverted over the course of the trial.
An assessment of compliance will be carried out looking in particular at repellent and placebo use and LLIN use. Adverse events in children under 12 years and in participants over 12 years will also be assessed.

## QUALITY CONTROL AND ASSURANCE

The use of the repellent and placebo lotion will be monitored by self-reporting in the monthly questionnaires. The amount of lotion left in the bottles will also monitored each month when they are returned for refilling, giving another indirect measure of lotion use. Lotion use will also be directly observed during evening ‘sniff checks’ carried out by the Project Manager.

## ETHICAL APPROVAL

Ethical Approval for this study was obtained from the Lao Ministry of Health and the Ethics Committee of the London School of Hygiene & Tropical Medicine.

## FINANCING AND INSURANCE

The trial is funded by a Gates Innovations Fund to PSI Laos.

## PUBLICATION POLICY

Results and methodology will be published in an open-access, peer-reviewed journal.

## REFERENCES

1. Antwi FB, Shama LM and Peterson RKD (2008) Risk assessments for the insect repellents DEET and picaridin. *Regulatory Toxicology and Pharmacology.* **51**: 31-6
2. Baimai V, Kijchalao U, Sawadwongporn P and Green CA (1988) Geographic distribution and biting behaviour of four species of the *Anopheles dirus* complex (Diptera: Culicidae) in Thailand. *Southeast Asian Journal of Tropical Medicine and Public Health.* **19**: 151–61.
3. Barnard DR (2000) *Repellents and Toxicants for Personal Protection.* Global Collaboration for Development of Pesticides for Public Health (GCDPP). Available from: http://www.who.int/malaria/publications/atoz/who_cds_ whopes_gcdpp_2000_5/en/index.html. World Health Organization. Communicable Disease Control, Prevention and Eradication. WHO Pesticide Evaluation Scheme (WHOPES).
4. Chareonviriyaphap T, Prabaripai A, Bangs MJ, Aum-Aung B (2003) Seasonal abundance and blood feeding activity of *Anopheles minimus* Theobald (Diptera: Culicidae) in Thailand. *Journal of Medical Entomology.* **40**(6): 876-81.
5. Do MC, Nguyen THV, Le QT, Tran LC, Le NA, Nguyen XT and Cooper RD (2008) Identification of *Anopheles minimus* complex and related species in Vietnam. *Southeast Asian Journal of Tropical Medicine and Public Health.* **39**(5): 827-31
6. Durrheim DN and Govere JM (2002) Malaria outbreak control in an African village by community application of 'deet' mosquito repellent to ankles and feet. *Medical and Veterinary Entomology.* **16**(1): 112-5.
7. Frances SP, Klein TA, Hildebrandt DW, Burge R, Noigamol C, Eikarat N, Sripongsai B and Wirtz RA (1996a) Laboratory and field evaluation of deet, CIC-4, and AI3-37220 against *Anopheles dirus* (Diptera: Culicidae) in Thailand. *Journal of Medical Entomology.* **33**(4): 511-5.
8. Frances SP, Easmsila C, Pilakasirp C and Linthicum KJ (1996b) Effectiveness of repellent formulations containing DEET against mosquitoes in northeastern Thailand. *Journal of the American Mosquito Control Association.* **12**(2): 331-3.
9. Frances SP and Wirtz RA (2005) Repellents: past, present, and future. *Journal of the American Mosquito Control Association.* **21**(Supplement 4): 1-3.
10. Gupta RK and Rutledge LC (1994) Role of repellents in vector control and disease prevention. *American Journal of Tropical Medicine and Hygiene.* **50**(Supplement 6): 82-6.
11. Hassan AA, Rahman WA, Rashid MZ, Shahrem MR, Adanan CR (2001) Composition and biting activity of *Anopheles* (Diptera: Culicidae) attracted to human bait in a malaria endemic village in peninsula Malaysia near the Thailand border. *Journal of Vector Ecology.* **26**(1): 70-5.
12. Hill N, Lenglet A, Arnéz AM and Carneiro I (2007) Plant based insect repellent and insecticide treated bed nets to protect against malaria in areas of early evening biting vectors: double blind randomised placebo controlled clinical trial in the Bolivian Amazon. *British Medical Journal.* **335**(7628): 1023-6.
13. Kroeger A, Gerhardus A, Kruger G, Mancheno M and Pesse A (1997) The contribution of repellent soap to malaria control. American *Journal of Tropical Medicine and Hygiene.* **56**(5): 580-4.
14. Lindsay SW, Ewald JA, Samung Y, Apiwathnasorn C and Nosten F (1998) Thanaka (*Limonia acidissima*) and deet (di-methyl benzamide) mixture as a mosquito repellent for use by Karen women. *Medical and Veterinary Entomology.* **12**: 295–301.
15. McGready R, Simpson JA, Htway M, White NJ, Nosten F and Lindsay SW (2001a) A double-blind randomized therapeutic trial of insect repellents for the prevention of malaria in pregnancy. *Transactions of the Royal Society of Tropical Medicine and Hygiene.* **95**(2): 137-8.
16. McGready R, Hamilton KA, Simpson JA, Cho T, Luxemburger C, Edwards R, Looareesuwan S, White NJ, Nosten F and Lindsay SW (2001b) Safety of the insect repellent *N,N*-diethyl-*M­*-toluamide (DEET) in pregnancy. *American Journal of Tropical Medicine and Hygiene.* **65**(4): 285-9
17. Moore SJ, Davies CR, Hill N and Cameron MM (2007) Are mosquitoes diverted from repellent-using individuals to non-users? Results of a field study in Bolivia. *Tropical Medicine and International Health.* **12**(4): 532-9.
18. Oo T (2003) *The biology and vector competence of the anopheline mosquitoes of Myanmar with special consideration of Anopheles dirus.* PhD Thesis. Combined Faculties for the Natural Sciences and for Mathematics, Ruperto-Carola University of Heidelberg, Germany.
19. Osimitz TG and Grothaus RH (1995) The present safety assessment of DEET. *Journal of the American Mosquito Control Association.* **11**(2): 274-8.
20. Qiu H, Jun HW and McCall JW (1998) Pharmacokinetics, formulation and safety of insect repellent *N,N*-diethyl-3-methylbenzamide (DEET): A review. *Journal of the American Mosquito Control Association.* **14**(1): 12-27.
21. Rattanarithikul R, Konishi E and Linthicum K (1996) Observations on nocturnal biting activity and host preference of anophelines collected in southern Thailand*. Journal of the American Mosquito Control Association.* **12**(1): 52-7.
22. Rowland M, Freeman T, Downey G, Hadi A and Saeed M (2004a) DEET mosquito repellent sold through social marketing provides personal protection against malaria in an area of all-night mosquito biting and partial coverage of insecticide-treated nets: a case–control study of effectiveness. *Tropical Medicine and International Health.* **9**(3): 343-50.
23. Rowland M, Downey G, Rab A, Freeman T, Mohammad N, Rehman H, Durrani N, Reyburn H, Curtis C, Lines J and Fayaz M (2004b) DEET mosquito repellent provides personal protection against malaria: a household randomized trial in an Afghan refugee camp in Pakistan. *Tropical Medicine and International Health.* **9**(3): 335-42.
24. Schultz G (1992) Biting activity of mosquitos (Diptera: Culicidae) at a malarious site in Palawan, Republic of the Philippines. *Southeast Asian Journal of Tropical Medicine and Public Health.* **23**(3): 464-9.
25. Selim S, Hartnagel RE Jr, Osimitz TG, Gabriel KL and Schoenig GP (1995) Absorption, Metabolism, and Excretion of *N,N* -Diethyl-*m*-toluamide Following Dermal Application to Human Volunteers. *Fundamental and Applied Toxicology* **25**: 95-100.
26. Singhasivanon P, Kidson C and Supavej S (2003) Mekong Malaria II: Update of malaria, multi-drug resistance and economic development in the Mekong region of southeast Asia. *Southeast Asian Journal of Tropical Medicine and Public Health.* **34**(Supplement 4): 1-102.
27. Socheath S, Seng C, Rath TS, Deesin V, Deesin T and Apiwathanasorn C (2000) Study on bionomics of principal malaria vectors in Kratie Province, Cambodia. *Southeast Asian Journal of Tropical Medicine and Public Health.* **31**(Supplement 1): 106-10.
28. Sungvornyothin S, Muenvorn V, Garros C, Manguin S, Prabaripai A, Bangs MJ and Chareonviriyaphap T (2006) Trophic behavior and biting activity of the two sibling species of the *Anopheles minimus* complex in western Thailand. *Journal of Vector Ecology.* **31**(2): 252-61.
29. Thavara U, Tawatsin A, Chompoosri J, Suwonkerd W, Chansang UR and Asavadachanukorn P (2001) Laboratory and field evaluations of the insect repellent 3535 (ethyl butylacetylaminopropionate) and deet against mosquito vectors in Thailand. *Journal of the American Mosquito Control Association.* **17**(3): 190-5.
30. Toma T, Miyagi I, Okazawa T, Kobayashi J, Saita S, Tuzuki A, Keomanila H, Nambanya S, Phompida S, Uza M, Takakura M (2002) Entomological surveys of malaria in Khammouane Province, Lao PDR, in 1999 and 2000. *Southeast Asian Journal of Tropical Medicine and Public Health.* **33**(3): 532-46.
31. Torres EP, Salazar NP, Belizario VY and Saul A (1997) Vector abundance and behaviour in an area of low malaria endemicity in Bataan, the Philippines. *Acta Tropica.* **63**: 209-20.
32. Trung HD, Bortel WV, Sochantha T, Keokenchanh K, Briët OJ and Coosemans M (2005) Behavioural heterogeneity of *Anopheles* species in ecologically different localities in Southeast Asia: a challenge for vector control. *Tropical Medicine and International Health.* **10**(3): 251-62.
33. Tun-Lin W, Thu MM, Than SM, Mya MM (1995) Hyperendemic malaria in a forested, hilly Myanmar village. *Journal of the American Mosquito Control Association.* **11**(4): 401-7.
34. Van Bortel W, Trung HD, Manh ND, Roelants P, Verlé P and Coosemans M (1999) Identification of two species within the *Anopheles minimus* complex in northern Vietnam and their behavioural divergences. *Tropical Medicine and International Health.* **4**(4): 257-65.
35. Vythilingam I, Phetsouvanh R, Keokenchanh K, Yengmala V, Vanisaveth V, Phompida S and Hakim SL (2003) The prevalence of *Anopheles* (Diptera: Culicidae) mosquitoes in Sekong Province, Lao PDR in relation to malaria transmission. *Tropical Medicine and International Health.* **8**(6): 525-35.
36. Vythilingam I, Sidavong B, Chan ST, Phonemixay T, Vanisaveth V, Sisoulad P, Phetsouvanh R, Hakim SL and Phompida S (2005) Epidemiology of malaria in Attapeu Province, Lao PDR in relation to entomological parameters. *Transactions of the Royal Society of Tropical Medicine and Hygiene.* **99**(11): 833-9.
37. WHO (2009) *Mortality and burden of disease estimates for WHO member states in 2004.* Available from: http://www.who.int/healthinfo/global_burden_disease/estimates_country/en/index.html. Department of Measurement and Health Information, World Health Organzation, Geneva.
38. WHO (2005) *Lao People’s Democratic Republic Country Profile.* Available from: http://rbm.who.int/wmr2005/profiles/laos.pdf. Roll Back Malaria Monitoring and Evaluation, World Health Organization, Geneva.
39. UNDP (2001) *National Human Development Report Lao PDR 2001. Advancing Rural Development.* Available from: http://www.undplao.org/whatwedo/factsheets/humandev/nhdr%20final.pdf. United Nations Development Programme, Lao PDR.
40. USEPA (1998) *Reregistration Eligibility Decision (RED) DEET*. Available from: http://www.epa.gov/oppsrrd1/ REDs/0002red.pdf. United States Environment Protection Agency. Office of Prevention, Pesticides and Toxic Substances, Washington DC.
41. Veltri JC, Osimitz TG, Bradford DC and Page BC (1994) Retrospective analysis of calls to Poison Control Centers Resulting from exposure to the insect repellent *N,N*-diethyl-*M*-toluamide (DEET) from 1985-1989. *Clinical Toxicology.* **32**(1): 1-16.
42. Yap HH, Jahangir K and Zairi J (2000) Field efficacy of four insect repellent products against vector mosquitoes in a tropical environment. *Journal of the American Mosquito Control Association.* **16**(3): 241-4.
43. Zhou H (2003) *Sporozoite rates of malaria vectors in the south of Yunnan, P.R. China.* MSc Thesis. London School of Hygiene & Tropical Medicine.
