# Supplementary material for: Can Topical Insect Repellents Reduce Malaria? A Cluster-Randomised Controlled Trial of the Insect Repellent N,N-diethyl-m-toluamide (DEET) in Lao PDR
Source: PLoS One. 2013 Aug 14;8(8):e70664. doi: 10.1371/journal.pone.0070664 (PMC3743820; doi:10.1371/journal.pone.0070664)
Supplement: Analysis S1 — Poisson Regression Analysis. (DOC) [file pone.0070664.s003.doc]

**Analysis S1: Poisson Regression Analysis**

Age, LLIN use, nights spent away from the village and self-reported lotion use were not included in any models as they were not significantly related to the outcomes in univariate analysis. The second socio-economic score was drop from the final models as it showed no effect. After accounting for socio-economic scores, gender and volume of lotion used there was no difference between the placebo and repellent arms for overall malaria incidence (rate ratio 0.99, 95% C.I. 0.62-1.59, p=0.967, Table 5.7) or *P. falciparum* incidence (rate ratio 0.94, 95% C.I. 0.56-1.59, p=0.815). *P. vivax* incidence was too low to enter a regression. A higher PCA1 score was associated with a reduced malaria risk in both models (overall malaria p=0.001; *P. falciparum* p=0.004). PCA3 increased the risk of any malaria infection although the significance was borderline. Being female decreased the risk of *P. falciparum* infection by almost 50% (risk ratio 0.53, 95% C.I. 0.31-0.91, p=0.021). The volume of lotion used irrespective of whether it was repellent or placebo was associated with a decrease in malaria risk, a household using all of their lotion had a 75% reduction in malaria compared to a household who used none (overall malaria: rate ratio 0.25, 95% C.I. 0.12-0.54, p<0.001; *P. falciparum*: rate ratio 0.17, 95% C.I. 0.07-0.39, p<0.001).

Table S1. Incidence risk ratios and 95% confidence intervals from ITT Poisson regressions on counts of overall malaria and *P. falciparum* infections.

| Variable included in model | | | Overall malaria | *P. falciparum* |
| --- | --- | --- | --- | --- |
| Treatment group | | Placebo | 1 | 1 |
| Repellent | | | 0.99 (0.62-1.59), p=0.967 | 0.94 (0.56-1.59), p=0.815 |
| Gender | Male | | 1 | 1 |
| Female | | | 0.63 (0.39-1.02), p=0.062 | 0.53 (0.31-0.91), p=0.021 |
| PCA1 | | | 0.76 (0.64-0.90), p=0.001 | 0.76 (0.63-0.92), p=0.004 |
| PCA3 | | | 1.23 (1.00-1.52), p=0.048 | - |
| Volume of lotion used | | | 0.25 (0.12-0.54), p<0.001 | 0.17 (0.07-0.39), p<0.001 |
| Observations | | | 43,960 | 43,960 |
| Model χ^2^ p-value | | | <0.001 | <0.001 |
| Variables excluded from model | | | PCA2, age, LLIN use,  Nights away from the village,  Self-reported lotion use | PCA2, PCA3, age, LLIN use  Nights away from the village,  Self-reported lotion use |
