# Supplementary material for: Can Topical Insect Repellents Reduce Malaria? A Cluster-Randomised Controlled Trial of the Insect Repellent N,N-diethyl-m-toluamide (DEET) in Lao PDR
Source: PLoS One. 2013 Aug 14;8(8):e70664. doi: 10.1371/journal.pone.0070664 (PMC3743820; doi:10.1371/journal.pone.0070664)
Supplement: Analysis S2 — Principal Components Analysis. (DOC) [file pone.0070664.s004.doc]

**Analysis S2: Principal Components Analysis**

Household socioeconomic data from the baseline survey was combined using a principal components analysis (PCA). Cooking fuel, radio ownership and goat ownership were excluded from the PCA as they did not contain sufficient variation between the trial households. The PCA included the occupation and years of education of the head of household, housing materials, electricity and water supply, ownership of motorbikes, televisions and tractors, pre-trial ownership of bed nets and ownership of buffalo, pigs, chickens and dogs. Over 95% of all heads of household were farmers and the median years of education was 3 (Table S2). About two thirds of houses had a metal roof and a similar number were made from wood rather than bamboo. Sixty percent of households had no electricity supply and about half had access to a pump for water. Motorbike ownership was most frequent, with about 40% of households owning at least one. About 60% of households also owned buffalo or pigs.

Three standardised scores for each household were created, PCA1, PCA2 and PCA3, the mean score for each treatment arm was compared by t-test and no significant differences found (PCA1: t=-0.77, p=0.442; PCA2: t=-0.99, p=0.322; PCA3: t=0.89, p=0.374; Table S2). PCA1 was created from almost all variables and provided a general socio-economic score. PCA2 was more heavily influenced by animal ownership and PCA3 was largely related to job and education of the head of household. Due to missing data PCA scores could only be created for 1,400 (87.7%) households. (Table S2)

Table S2. Household socio-economic data and resulting PCA scores per treatment arm.

| Socio-economic variable | Repellent | Placebo |
| --- | --- | --- |
| % of heads of household who are farmers | 97.0 | 95.9 |
| Median years of education of head of household (IQR) | 3 (1-5) | 3 (1-5) |
| % of houses made of bamboo | 34.3 | 35.5 |
| % of houses made of wood | 66.3 | 66.8 |
| % of houses with a metal roof | 68.6 | 68.7 |
| % of households with no electricity supply | 59.0 | 61.7 |
| % of households with access to a water pump | 51.6 | 52.1 |
| % of households that use wood as primary cooking fuel | 97.5 | 97.0 |
| % of households that own at least one motorbike | 38.7 | 41.3 |
| % of households that own a tractor | 16.2 | 18.8 |
| % of households that own a radio | 19.8 | 19.7 |
| % of households that own a television | 23.2 | 24.3 |
| % of households owning ITNs prior to the trial | 86.3 | 85.5 |
| % of households owning buffalo | 56.7 | 58.4 |
| % of households owning pigs | 57.4 | 60.1 |
| % of households owning goats | 11.2 | 11.4 |
| % of households owning chickens | 70.4 | 73.4 |
| % of households owning dogs | 47.2 | 46.3 |
| Mean PCA1 score (95% C.I.) | 0.04 (-0.08, 0.17) | 0.11 (-0.01, 0.24) |
| Mean PCA2 score (95% C.I.) | -0.01 (-0.10, 0.07) | 0.05 (-0.05, 0.15) |
| Mean PCA3 score (95% C.I.) | 0.01 (-0.07, 0.10) | -0.04 (-0.13, 0.04) |
